# Efficacy and Safety of Low-Dose Peginterferon Alpha-2a Plus Ribavirin on Chronic Hepatitis C

**DOI:** 10.1155/2012/302093

**Published:** 2012-12-03

**Authors:** Zehui Yan, Ke Fan, Xiaohong Wang, Qing Mao, Guohong Deng, Yuming Wang

**Affiliations:** Institute of Infectious Diseases, Southwest Hospital, The Third Military Medical University, Chongqing 400038, China

## Abstract

*Background*. The purpose of this study was to assess the efficacy and safety of low-dose peg-IFN **α**-2a plus ribavirin on the treatment of patients with chronic hepatitis C virus (HCV) infection. *Patients and Methods*. A total of 243 HCV patients treated with different doses of peg-IFN **α**-2a plus ribavirin were stratified into three groups. End-of-treatment response (ETR) and sustained viral response (SVR) were evaluated for efficacy. Adverse events and laboratory abnormalities were conducted for safety. *Results*. ETR and SVR in group I were obtained in 83.9% and 68.9% of the patients, separately, which was similar to groups II (84.1% and 68.3%) and III (81.7% and 66.7%). The received peg-IFN **α**-2a dose was not the independent factor-related SVR in our population (OR, 1.31; 95% CI, 0.94–1.81; *P* = 0.106). The frequency of no adverse events reported in group III (24.7%) was significantly higher than that in group I (11.5%) and group II (12.7%) (*P* = 0.036). *Conclusions*. The peg-IFN **α**-2a 90 **μ**g/week plus ribavirin is as effective as, and better tolerated than, peg-IFN **α**-2a standard dose with ribavirin in the treatment of chronic hepatitis C. This low-dose combination achieves high SVR rates and may be cost-saving.

## 1. Background

The hepatitis C virus (HCV) is a major public health problem affecting 170 million people worldwide, which can result in progressive hepatic injury and fibrosis, culminating in cirrhosis and end-stage liver disease [1, 2]. The currently recommended therapy for patients with chronic hepatitis C is the combination of a pegylated interferon (peg-IFN) alfa plus ribavirin [3]. There are two licensed peg-IFN: peginterferon alfa-2b (peg-IFN *α*-2b) and peginterferon alfa-2a (peg-IFN *α*-2a) [4]. The doses of these two forms of peg-IFN differ. According to the updated practice guideline, the optimal dose of peg-IFN *α*-2b is 1.5 *μ*g/kg/week dosed according to body weight, while peg-IFN *α*-2a is administered at a fixed dose of 180 *μ*g weekly given subcutaneously together with weight-based ribavirin from 1,000 to 1,200 mg daily for treatment of chronic hepatitis C [3].

The standard therapy regimen with recommended dose peg-IFN in combination with ribavirin is effective in chronic hepatitis patients, resulting in sustained virologic response (SVR) in 40–50% of patients with genotype 1, and around 80% in those infected with genotype 2/3 [3, 5]. However, these peg-IFN-based regimens are accompanied by many adverse effects, including fever, depression, anemia, neutropenia, and thrombocytopenia, and many patients treated require reductions in the dose of one or both agents, or must be discontinued from treatment, because of adverse events [6–8]. The peg-IFN-based combination therapy is also associated with significant cost, which is major limiting factor in initiating treatment and a major reason of treatment withdrawal during the course of therapy, especially for the patients in developing countries [9, 10]. Therefore, it is rational to hypothesize that lower doses of peg-IFN might reduce the cost of therapy, decrease adverse events, and increase the likelihood of completing treatment and achieving an SVR.

Use of lower doses of peg-IFN might be prudent. Some studies have evaluated the effect of reduced dose peg-IFN *α*-2b (lowered from 1.5 mg/kg body weight to 0.5 mg/kg body weight) plus ribavirin on chronic hepatitis C and found that low-dose peg-IFN *α*-2b in combination with ribavirin is as effective as standard high-dose peg-IFN *α*-2b plus ribavirin on chronic hepatitis C [11–14]. A prospective, randomized, open-label, single-center trial has suggested that peg-IFN *α*-2a plus ribavirin produced a significantly higher SVR rate than peg-IFN *α*-2b plus ribavirin in patients with chronic HCV infection [15]. Therefore, it would be worthwhile to study whether the dose of peg-IFN *α*-2a could be lowered from 180 *μ*g/week to 90 *μ*g/week in combination with standard dose of ribavirin without compromising the efficacy of therapy in chronic hepatitis C patients.

Nevertheless, there is limited data on efficacy of low-dose peg-IFN *α*-2a (90 *μ*g/week) plus ribavirin on chronic hepatitis C. In a previous study, forty-eight weeks of treatment with 90 *μ*g/week peg-IFN *α*-2a is safe and produces an SVR in 35–40% of patients with chronic hepatitis C [16]. However, the enrolled patients in that study was individuals with chronic hepatitis C and end-stage renal disease who were being treated with hemodialysis, the treatment regimen in that study was given peg-IFN *α*-2a monotherapy without ribavirin. In recent years, some patients with chronic hepatitis C have been treated with low-dose peg-IFN *α*-2a (90 *μ*g/week) plus ribavirin under the guidance of physicians at Southwest hospital. The aims of this study were to assess the efficacy and safety of peg-IFN *α*-2a at doses of 90 *μ*g/week, 135 *μ*g/week, and 180 *μ*g/week plus ribavirin on chronic hepatitis C patients.

## 2. Patients and Methods

### 2.1. Study Participants

This study was a per protocol study and approved by the ethical committee of Southwest Hospital, Chongqing, China. There is prospective informed consent to all participants and prospective baseline characteristics collecting. We identified all consecutive patients with chronic hepatitis C, who were registered and followed up at Southwest Hospital from January 2004 to June 2011. The patients aged 18–65 years who completed the proposed duration of antiviral therapy with peg-IFN *α*-2a plus ribavirin and had at least 24 weeks followup period after the end of treatment were included. The diagnosis of chronic HCV was made by a persistent or intermittent elevation of alanine aminotransferase (ALT) over a 6-month period and detection of HCV-RNA in the sera. Patients meeting with the following criteria were excluded: (i) coinfection with human immunodeficiency virus or hepatitis B virus, (ii) peripheral blood leucocyte count <3 × 10^9^/L or platelet count <70 × 10^9^/L or hemoglobin level lower than 100 g/L, (iii) alcohol intake exceeding 20 g/day or presence of drug abuse, (iv) thyroid dysfunction, (v) pregnancy and lactation (vi) presence of decompensated cirrhosis and liver disease due to other etiology such as autoimmune or drug-induced hepatitis, and (vii) concomitant serious concurrent medical illnesses, such as malignancy, severe cardiopulmonary disease, or uncontrolled diabetes mellitus. All patients underwent routine hematological investigations, liver function test, and abdominal ultrasound examination. The level of serum HCV RNA and the HCV genotype were determined by nucleotide sequencing of the core-envelope 1 region as described in our previous research [17].

According to the different dose of peg-IFN *α*-2a suggested by local physicians, the patients of current study were stratified into three groups as follows: group I, which was treat with 180 *μ*g subcutaneous weekly dose of peg-IFN *α*-2a plus ribavirin, group II, which was administered a dose of 135 *μ*g/week of peg-IFN *α*-2a plus ribavirin, and group III, which received 90 *μ*g per week of peg-IFN *α*-2a plus ribavirin. The dosage of ribavirin was determined by body weight (800 mg/day in patients < 60 kg; 1000 mg/day day in patients ≥ 60 kg). The durations of treatment were 48 wks for patients infected with HCV genotype 1, and 24 wks for patients infected with HCV genotype 2/3/6.

### 2.2. Efficacy Assessments

Serum HCV RNA levels, routine hematological workup and biological function were determined at baseline, then at weeks 12, 24, and 48 during treatment. Following the completion of treatment, all patients were evaluated at 12 and 24 wk. On each visit during followup, side effects of the therapy were carefully recorded and relevant investigations were performed when necessary. End-of-treatment response (ETR) and SVR were defined, respectively, as a negative qualitative HCV RNA level at the end of treatment and after 24 weeks of untreated followup. Early virological response (EVR) was defined as qualitative HCV RNA negative (complete EVR) or a reduction from baseline HCV RNA level of >2log⁡10 IU/mL at week 12 (partial EVR). All patients with detectable HCV RNA at week 24 stopped treatment and were classified as nonresponders. Virological relapse was defined as reversion to HCV RNA-positive status in a patient who had an undetectable HCV RNA level (<50 IU/mL) at the end of treatment. The primary efficacy end point of the study was SVR.

### 2.3. Safety Assessments

Safety assessments including adverse events, vital signs, and laboratory tests were conducted throughout treatment and followup. Patients were monitored at each outpatient visit for adverse events and laboratory abnormalities. Information on possible adverse events was obtained by questioning the patients in a structured way about specific, commonly observed, and expected side effects of the study medication, such as flu-like symptoms, fatigue, nausea/vomiting/diarrhoea, dizziness, depression, and hair loss. In addition, spontaneously reported adverse events were recorded.

### 2.4. Statistical Analysis

Statistical analysis was performed using SPSS software (version 9.0; SPSS Inc, Chicago, IL). Quantitative variables were expressed as mean ± standard deviation and were compared by the Mann-Whitney rank sum test. Frequencies were calculated for categorical variables and were compared by the *χ* ^2^ test. Multivariable logistic stepwise regression analysis was used to explore the independent effect of the treatment and the baseline factors (age, body weight sex, presence of cirrhosis, ALT level, HCV RNA level, HCV genotype) on the likelihood of achieving SVR, with the difference between groups reported with 95% confidence intervals (CIs). A 2-sided *P* value less than 0.05 was considered significant.

## 3. Results

### 3.1. Characteristics of the Patient

Between January 2004 and June 2011, a total of 243 patients treated with peg-IFN *α*-2a plus ribavirin were included in this analysis. The age of the total 243 patients ranged from 19 to 65 years with a mean age (±SD) of 39.8 ± 9.9 years. Of them, 145 patients (59.7%) were males and 98 patients (40.3%) were females, included 130 patients (53.5%) with HCV-1b infection, 20 patients (8.2%) with HCV-2a infection, 60 patients (24.7%) with HCV-3 infection, and 33 patients (13.6%) with HCV-6a infection. Of them, 66 individuals (27.2%) had evidence of obvious cirrhosis or extensive fibrosis at the time of beginning therapy. The pretreatment ALT levels were 97.5 ± 124.6 IU/L, and 96 patients (39.5%) had elevated ALT ≥2 times the upper limit of normal (ULN). The pretreatment HCV RNA levels of all patients ranged from 1 × 10^2^ to 1.7 × 10^7^ IU/mL with median 6.4 × 10^5^ IU/mL, and the pretreatment HCV RNA levels of 125 patients (51.4%) were higher than 6.0 × 10^5^ IU/mL.

There were 87 patients treated with peg-IFN *α*-2a 180 *μ*g/week plus ribavirin in group I, while 63 cases treated with peg-IFN *α*-2a 130 *μ*g/week plus ribavirin in group II, and 93 cases treated with peg-IFN *α*-2a 90 *μ*g/week plus ribavirin in group III. The baseline characteristics of the three groups are shown in Table 1. The major factors of demography and anthropometry (age, gender, and body weight) and clinical basic characteristics (cirrhosis, the pretreatment ALT, and HCV RNA levels) of the three treatment groups were similar (all *P* > 0.05).

### 3.2. Efficacy

Table 2 compares the virological response during treatment and at the end of followup to peg-IFN *α*-2a plus ribavirin therapy among patients with different peg-IFN *α*-2a dose groups. Except for complete EVR, differences in the rate of EVR, ETR, and SCR among patients treated with three different doses peg-IFN *α*-2a plus ribavirin all were not statistically significant (*P* > 0.05).

Among the 243 patients, only 172 patients were tested HCV RNA level at week 12 of treatment, among these 172 patients, 149 patients achieved EVR. The rate of RVR in group I (91.5%) was higher than that in group II (88.2%) and even higher than that in group I (82.3%), although the difference was not statistically significant (*P* = 0.197). The majority of patients obtained a complete EVR, while the number of those who obtained a partial EVR was only 11.0%, with no difference among the three groups. However, for the patients who achieved EVR, the rate of complete EVR in group III (64.5%) was significantly lower than that in group I (83.1%) and that in group II (80.4%) (*P* = 0.038).

Overall, ETR was seen in 202/243 patients (83.1%). The rates of ETR of three groups were 83.9% for group I, 84.1% for group II, and 81.7% for group III, respectively. There were no significantly differences of the ETR rates for the three groups (*P* = 0.899). At the end of 24 weeks followup, a SVR was obtained in 165 patients (67.9%), including 60 of 87 (68.9%) in group I, 43 of 63 (68.3%) in group II, and 62 of 93 (66.7%) in group III. There was no significant difference of SVR among the three independent groups with different peg-IFN *α*-2a dose (*P* = 0.945). The total number of patients who experienced a relapse during followup was 37 of 243 (15.2%), including 13 of 87 (14.9%) in group I versus 10 of 63 (15.9%) in group II and 14 of 93 (15.1%) in group III.

As reported in Table 2, 73 of 130 patients (56.2%) infected with genotype 1 achieved a SVR, including 29 of 50 (58.0%) in group I, 18 of 32 (56.3%) in group II, and 26 of 48 (54.2%) in group III (*P* = 0.877). A total of 66 of 80 patients with genotype 2 or 3 obtained a SVR. The SVR rates of patients infected with genotype 2 or 3 for the three groups with different peg-IFN *α*-2a dose were similar. The rate of SVR of patients infected with genotype 6 in group I (83.3%) was higher than that in group II (80.0%) and even higher than that in group I (72.7%), but the difference was not statistically significant (*P* = 0.819). Among patients without cirrhosis, an SVR was achieved by 130 of 177 patients (73.4%). The SVR rates of patients without cirrhosis for the three groups with different peg-IFN *α*-2a dose were similar. The rate of SVR of patients with cirrhosis in group I (47.5%) was lower than that in group II (55.6%) and even higher than that in group I (58.1%), although the difference was not statistically significant (*P* = 0.834). An SVR was obtained in 87 of 118 patients (73.7%) with a baseline HCV RNA level <600,000 IU/mL, including 29 of 40 (72.5%) in group I, 20 of 28 (71.4%) in group II, and 38 of 50 (76.6%) in group III. No significantly differences of the ETR rates was found among the three groups (*P* = 0.887). In 125 patients with higher baseline HCV RNA level (≥600,000 IU/mL), 78 cases achieved SVR (62.4%), including 31 of 47 (66.0%) in group I, 23 of 35 (65.7%) in group II, and 24 of 43 (55.8%) in group III.

To further analyze the influence of low-dose peg-IFN *α*-2a treat regimen to SVR, we also conducted multivariate stepwise analysis included in the model the following preselected variables: peg-IFN *α*-2a dose of treatment, gender, age, HCV genotype, absence of cirrhosis, and HCV RNA level. As Table 3 showed, according to multivariate stepwise analysis, SVR was significantly associated with HCV genotype 2/3/6 (OR, 3.87; 95% CI, 2.16–5.89; *P* = 0.0002), absence of cirrhosis (OR, 2.47; 95% CI, 1.36–3.671; *P* = 0.008), and lower (<600 IU/mL × 10^3^) HCV RNA level (OR, 1.68; 95% CI, 1.12–2.57; *P* = 0.038). However, the peg-IFN *α*-2a dose of treatment was not the independent factor related SVR in our population (OR, 1.31; 95% CI, 0.94–1.81; *P* = 0.106).

### 3.3. Safety

Treatment was generally well tolerated with no unexpected safety concerns. No serious adverse events (death or any kind of life-threatening event) were reported. As Table 4 showed, a total of 47 patients had a hemoglobin concentration <80 g/L, 6 patients (2.5%) had a neutrophil count <0.75 × 10^9^/L, and 9 patients (3.7%) had a platelet count <50 × 10^9^/L. There was no statistically significant difference for the incidence of the hematological toxicity among the three different peg-IFN *α*-2a dose groups (*P* > 0.05). However, only one patient of group III (1.1%) occurred the thyroid dysfunction during the treatment, while 9 patients of group I (10.2%) and 5 patients of group II (7.9%) occurred the thyroid dysfunction (*P* = 0.028).

Table 4 also showed the types and frequencies of common adverse events (>10%). Overall, 41 of 243 patients (16.9%) reported no adverse events, including 10 of 87 (11.5%) in group I, 8 of 63 (12.7%) in group II, and 23 of 93 (24.7%) in group III. The frequencies of common adverse events were similar between the group I and group II (all *P* > 0.05), although the frequencies in group II were slightly lower than that in group I. There was significant difference of adverse events frequencies between patients with peg-IFN *α*-2a 90 *μ*g/week plus ribavirin treatment than those with peg-IFN *α*-2a 180 *μ*g/week plus ribavirin treatment (*P* = 0.022). Furthermore, the frequencies of the top three common adverse events, fatigue, fever, and headache in group III were also significant lower than that in group I.

## 4. Discussion

The current standard of care for HCV infection, peg-IFN alfa plus ribavirin is costly and has a significant adverse event profile. It has been reported that reduced doses of ribavirin have been found to be effective and safe in the treatment of genotype 2 or 3 HCV infections [18]. It also has been found that 135 *μ*g peg-IFN *α*-2a weekly combined with RBV dosed daily according to body weight was sufficient for treatment of genotype 2 and 3 chronic hepatitis C [19], and 135 *μ*g peg-IFN *α*-2a weekly monotherapy also effective in genotype 1 patients with cirrhosis [20]. Nevertheless, there is a paucity data on efficacy and safety of low-dose peg-IFN *α*-2a (90 *μ*g/week) plus ribavirin on chronic hepatitis C. To our knowledge, our observational study is the first study to compare the efficacy and safety/tolerability of the combination therapy of low-dose peg-IFN a-2a (90 *μ*g/week) plus ribavirin in naïve patients with chronic HCV from China.

The main conclusion of our study can be summarized in two points. First, we observed that the rates of SVR and ETR in patients with treatment of peg-IFN *α*-2a 90 *μ*g/week plus ribavirin were all similar to those in patients with treatment of peg-IFN *α*-2a 180 *μ*g/week plus ribavirin. Second, current study suggested that the peg-IFN *α*-2a 90 *μ*g/week plus ribavirin was better tolerated than the peg-IFN *α*-2a 180 *μ*g/week and 135 *μ*g/week plus ribavirin, based on the fact that adverse events were less frequently reported with the lower peg-IFN *α*-2a dose. Although the difference in rates of complete EVR between patients with treatment of peg-IFN *α*-2a 90 *μ*g/week plus ribavirin and those with treatment of peg-IFN *α*-2a 180 *μ*g/week plus ribavirin was statistically significant, we think there are two possible reasons for this. (i) A total 71 of 243 patients (29.2%) missed the data of HCV RNA level at week 12 during treatment, which would cause false statistically significant about EVR. (ii) Similar to the peg-IFN *α*-2b [21], peg-IFN *α*-2a also dose-dependently correlated with complete EVR. However, SVR, representing the primary efficacy endpoint, did not significantly differ between the peg-IFN *α*-2a higher and lower dose. Furthermore, the peg-IFN *α*-2a dose of treatment was not the independent factor related SVR according to multivariate stepwise analysis.

A number of host and viral factors influence SVR rates in patients with chronic hepatitis C [22, 23]. The most important factors are the genotype of the infecting HCV strain and baseline HCV RNA level. Patient age, gender, race, body mass index, and the extent of hepatic fibrosis all influence SVR rates. Patients with the IL28B rs12979860 C/C genotype have significantly higher on-treatment response rates than patients carrying the T-allele [24]. The ribavirin dose used in the present study was lower than the frequently recommended (1000–1200 mg daily). Taking the average body weight of our Chinese population into account, the majority of patients in all treatment groups received a daily ribavirin dose of >10 mg/kg, which, by means of a retrospective subgroup analysis of Manns' phase III trial, has previously been suggested to be the most efficacious [6]. Thus, it seems highly unlikely that the results of our study were affected by the flat ribavirin dose used. Additionally, in our present study, SVR in our whole cohort was significantly associated with HCV genotype 2/3/6, absence of cirrhosis, and lower HCV RNA level (<600 IU/mL × 10^3^), However, the major factors of demography and anthropometry (age, gender, and body weight) and clinical basic characteristics (cirrhosis, the pretreatment ALT, and HCV RNA levels) of the three treatment groups with different peg-IFN *α*-2a dose were similar. Thus, it also seems unlikely that the results of our study were affected by the different baseline characteristics of three groups.

We think there are some other possible reasons that may be contributed to the similar effect of low-dose peg-IFN *α*-2a plus ribavirin on chronic hepatitis C. First, the body weight and body mass index were lower in our Chinese population. According to the results from China health and nutrition surveys [25]. The mean body weight of Chinese adults is 55.9 kg with mean body mass index 21.7 kg/m^2^. This lower body weight of patients in the present study may be in favor of achieving a higher SVR even when treatments with low-dose peg-IFN *α*-2a plus ribavirin. Second, the high frequency of the rs12979860 genotype CC in Chinese population also might explain why the SVR rate of patients treated with low-dose peg-IFN *α*-2a plus ribavirin is similar with that of patients treated with high-dose peg-IFN *α*-2a, and higher than that of the average global population. The rs12979860 genotype CC, which was associated with SVR, was the primary genotype (nearly 90%), and genotypes CT and TT were found in only about 10% individual within Chinese population [26, 27]. Third, adherence to peg-IFN regimen was reported to be an important factor for SVR [28]. A study by Sood et al. [11] also revealed that a low-dose peg-IFN regimen was associated with fewer grade 3 and 4 side-effects in neutrophil and platelet counts, a potential benefit that does not manifest when patients are treated with the standard high dose regimen. These observations are likely to improve the compliance of the patients. Reduced doses of peg-IFN may decrease cost and reduce the frequency and severity of side-effects, then increase tolerability and effancy.

In conclusion, our study is the first to indicate that the peg-IFN *α*-2a 90 *μ*g/week dose is as effective as peg-IFN *α*-2a 180 *μ*g/week when combined with ribavirin in the treatment of chronic hepatitis C, but suggesting a better tolerability of the peg-IFN *α*-2a 90 *μ*g/week plus ribavirin. However, as a per protocol analysis performed only in one centre, these findings need to be confirmed in larger, preferably double-blind trials, such as for example the study individualized dosing efficacy versus flat dosing to assess optimal peg-IFN *α*-2a therapy.

## Figures and Tables

**Table 1:** The baseline characteristics of the patients.

Characteristics	Overall (*n* = 243)	Group I: peg-IFN *α*-2a 180 *μ*g/week plus ribavirin (*n* = 87)	Group II: peg-IFN *α*-2a 135 *μ*g/week plus ribavirin (*n* = 63)	Group III: peg-IFN *α*-2a 90 *μ*g/week plus ribavirin (*n* = 93)
Demography and anthropometry				
Age (y), Mean (SD)	39.8 (9.9)	38.5 (8.8)	38.7 (9.7)	41.7 (10.7)
Sex, male/female (% male)	145/98 (59.7)	53/34 (60.9)	38/25 (60.3)	54/39 (58.1)
Mean (SD) body wt (kg)	59.2 (12.4)	62.5 (10.8)	61.3 (10.4)	57.3 (10.6)
Genotype, *n* (%)				
1	130 (53.5 )	50 (52.3)	32 (50.8)	48 (51.6)
2	20 (8.2%)	4 (4.6)	6 (9.4)	10 (10.8)
3	60 (24.7)	21 (24.1)	15 (23.8)	24 (25.8)
6	33 (13.6 )	12 (13.8)	10 (15.9)	11 (11.8)
Diagnosis, *n* (%)				
Chronic hepatitis	177 (72.8)	66 (75.9)	45 (71.4)	66 (71.0)
Cirrhosis	66 (27.2)	21 (24.1)	18 (28.6)	27 (29.0)
ALT				
Mean (SD) ALT level, IU / L,	97.5 (124.6)	112.1 (164.3)	104.2 (178.1)	92.1 (152.7)
No. (%) with <2 ULN	147 (60.5)	56 (64.4)	39 (61.9)	52 (55.9)
No. (%) with ≥2 ULN	96 (39.5)	31 (35.6)	24 (38.1)	41 (44.1)
HCV RNA level				
Median (range) HCV RNA, IU/mL × 10^3^	636 (0.10–17650)	678 (0.50–17650)	625 (0.20–14200)	512 (0.10–16250)
No. (%) with <600 IU/mL × 10^3^	118 (48.6)	40 (46.0)	28 (44.4)	50 (53.8)
No. (%) with ≥600 IU/mL × 10^3^	125 (51.4)	47 (54.0)	35 (55.6)	43 (46.2)

Notes. SD: standard deviation, ALT: serum alanine aminotransferase levels; ULN: upper limit of normal.

**Table 2:** Virological response during treatment and at the end of followup.

Virological Response	Overall (*n* = 243)	Group I: peg-IFN *α*-2a 180 *μ*g/week plus ribavirin (*n* = 87)	Group II: peg-IFN *α*-2a 135 *μ*g/week plus ribavirin (*n* = 63)	Group III: peg-IFN *α*-2a 90 *μ*g/week plus ribavirin (*n* = 93)	*P* value
EVR, no./total (%)*	149/172 (86.7)	54/59 (91.5)	45/51 (88.2)	50/62 (82.3)	0.197
Complete	130/172 (75.6)	49/59 (83.1)	41/51 (80.4)	40/62 (64.5)	0.038
Partial	19/172 (11.0)	5/59 (8.5)	4/51 (7.8)	10/62 (16.1)	0.278
ETR, no./total (%)	202/243 (83.1)	73/87 (83.9)	53/63 (84.1)	76/93 (81.7)	0.899
SVR, no./total (%)	165/243 (67.9)	60/87 (68.9)	43/63 (68.3)	62/93 (66.7)	0.945
1b	73/130 (56.2)	29/50 (58.0)	18/32 (56.3)	26/48 (54.2)	0.877
2/3	66/80 (82.5)	21/25 (84.0)	17/21 (81.0)	28/34 (82.4)	0.964
6a	26/33 (78.8)	10/12 (83.3)	8/10 (80%)	8/11 (72.7)	0.819
SVR by diagnosis, no./total (%)					
Chronic hepatitis	130/177 (73.4)	50/66 (75.8)	33/45 (73.3)	47/66 (71.2)	0.839
Cirrhosis	35/66 (53.0)	10/21 (47.6)	10/18 (55.6)	15/27 (58.1)	0.834
SVR by baseline HCV RNA, no. /total (%)					
<600,000 IU/mL	87/118 (73.7)	29/40 (72.5)	20/28 (71.4)	38/50 (76.0)	0.887
≥600,000 IU/mL	78/125 (62.4)	31/47 (66.0)	23/35 (65.7)	24/43 (55.8)	0.545

Notes. *Among the 243 patients who completed the proposed duration of antiviral therapy with peg-IFN *α*-2a plus ribavirin and had at least 24 weeks followup period after the end of treatment, only 172 patients were tested HCV RNA level at week 12 of treatment, among these 172 patients, 149 patients achieved RVR. *P* value was given by 2 × 3 chi-square test comparing the three different peg-IFN *α*-2a dose groups. EVR: early virological response. ETR: end-of-treatment response. SVR: sustained virologic response.

**Table 3:** Results of multivariate stepwise analysis for associated factor to SVR.

Factor	Category	Patients with SVR, no./total (%)	Patients without SVR, no./total (%)	*P* value	95% CI
Gender					
	Male	98/145 (67.6)	47/145 (33.4)	0.114	1.23 (0.91–2.03)
	Female	67/98 (68.4)	31/98 (31.6)		
Age					
	<40 years	93/132 (70.5)	39/132 (29.5)	0.093	1.02 (0.83–1.72)
	≥40 years	74/111 (66.7)	37/111 (33.3)		
HCV genotype					
	Genotype 1	73/130 (56.2)	57/130 (43.8)	0.0002	3.87 (2.16–5.89)
	Genotype 2/3/6	92/113 (81.4)	21/113 (18.6)		
Absence of cirrhosis					
	With	35/66 (53.0)	31/66 (47.0)	0.008	2.47 (1.36–3.67)
	Without	130/177 (73.4)	47/177 (26.5)		
HCV RNA level					
	<600 IU/mL × 10^3^	87/118 (73.7)	31/118 (26.3)	0.038	1.68 (1.12–2.57)
	≥600 IU/mL × 10^3^	78/125 (62.4)	47/125 (37.6)		
Peg-IFN *α*-2a dose					
	90 *μ*g/week	62/93 (66.7)	31/93 (33.3)	0.106	1.31 (0.94–1.81)
	180 and135 *μ*g/week	103/150 (68.7)	47/150 (31.3)		

Notes. OR: odds ratio; CI: confidence interval. The association was analyzed by multivariate stepwise analysis included in the model the following preselected variables: peg-IFN *α*-2a dose of treatment (lower dose versus standard dose), gender (male versus female), age (aged < 40 years versus ≥ 40 years), HCV genotype (genotype 1 versus 2/3/6), absence of cirrhosis(with versus without), and HCV RNA level (<600 IU/mL × 10^3^ versus ≥600 IU/mL × 10^3^).

**Table 4:** Adverse clinical and laboratory reported during treatment and followup.

Adverse Events	Overall (*n* = 243)	Group I: peg-IFN *α*-2a 180 *μ*g/week plus ribavirin (*n* = 87)	Group II: peg-IFN *α*-2a 135 *μ*g/week plus ribavirin (*n* = 63)	Group III: peg-IFN *α*-2a 90 *μ*g/week plus ribavirin (*n* = 93)	*P* value
Laboratory abnormalities					
Hemoglobin < 80 g/L	47 (19.3)	18 (20.7)	12 (19.1)	17 (18.3)	0.918
Neutrophils < 0.75 × 10^9^/L	6 (2.5)	3 (3.4)	2 (2.3)	1 (1.1)	0.541
Platelets < 50 × 10^9^/L	9 (3.7)	4 (4.6)	3 (4.7)	2 (2.2)	0.600
Thyroid dysfunction	15 (6.2)	9 (10.2)	5 (7.9)	1 (1.1)	0.028
No adverse events, *n* (%)	41 (16.9)	10 (11.5)	8 (12.7)	23 (24.7)	0.036
Common adverse events (>10%)					
Fatigue	154 (63.4)	65 (74.7)	38 (60.3)	51 (54.8)	0.018
Fever	134 (55.1)	55 (63.2)	34 (54.0)	45 (48.4)	0.132
Headache	67 (27.6)	31 (35.6)	16 (25.4)	20 (21.5)	0.096
Dermatitis or Pruritus	46 (18.9)	20 (22.9)	11 (17.5)	15 (16.1)	0.473
Nausea	34 (13.9)	15 (17.2)	9 (14.3)	10 (10.8)	0.454
Arthralgia or Myalgia	30 (12.3)	12 (13.8)	9 (14.3)	9 (9.7)	0.607
Irritability or Depression	27 (11.1)	11 (12.6)	8 (12.7)	8 (8.6)	0.619
Insomnia	25 (10.3)	10 (11.5)	8 (12.7)	7 (7.5)	0.522

Notes. All values are expressed as *n* (%). *P* value was given by 2 × 3 chi-square test comparing the three different peg-IFN *α*-2a dose groups.

## References

[1] Shepard CW, Finelli L, Alter MJ (2005). Global epidemiology of hepatitis C virus infection. *The Lancet Infectious Diseases*.

[2] Negro F, Alberti A (2011). The global health burden of hepatitis C virus infection. *Liver International*.

[3] Ghany MG, Strader DB, Thomas DL, Seeff LB (2009). Diagnosis, management, and treatment of hepatitis C: an update. *Hepatology*.

[4] Zeuzem S, Welsch C, Herrmann E (2003). Pharmacokinetics of peginterferons. *Seminars in Liver Disease*.

[5] McHutchison JG, Lawitz EJ, Shiffman ML (2009). Peginterferon alfa-2b or alfa-2a with ribavirin for treatment of hepatitis C infection. *The New England Journal of Medicine*.

[6] Manns MP, McHutchison JG, Gordon SC (2001). Peginterferon alfa-2b plus ribavirin compared with interferonalfa-2b plus ribavirin for initial treatment of chronic hepatitis C: a randomised trial. *The Lancet*.

[7] Sokal EM, Bourgois A, Stéphenne X (2010). Peginterferon alfa-2a plus ribavirin for chronic hepatitis C virus infection in children and adolescents. *Journal of Hepatology*.

[8] Fried MW, Shiffman ML, Reddy KR (2002). Peginterferon alfa-2a plus ribavirin for chronic hepatitis C virus infection. *The New England Journal of Medicine*.

[9] Solomon M, Bonafede M, Pan K (2011). Direct medical care costs among pegylated interferon plus ribavirin-treated and untreated chronic hepatitis C patients. *Digestive Diseases and Sciences*.

[10] McCombs JS, Yuan Y, Shin J, Saab S (2011). Economic burden associated with patients diagnosed with hepatitis C. *Clinical Therapeutics*.

[11] Sood A, Midha V, Hissar S (2008). Comparison of low-dose pegylated interferon versus standard high-dose pegylated interferon in combination with ribavirin in patients with chronic hepatitis C with genotype 3: an Indian experience. *Journal of Gastroenterology and Hepatology*.

[12] Meyer-Wyss B, Rich P, Egger H (2006). Comparison of two PEG-interferon alpha-2b doses (1.0 or 1.5 *μ*g/kg) combined with ribavirin in interferon-naïve patients with chronic hepatitis C and up to moderate fibrosis. *Journal of Viral Hepatitis*.

[13] Gupta R, Ramakrishna CH, Lakhtakia S, Tandan M, Banerjee R, Reddy DN (2006). Efficacy of low dose peginterferon alpha-2b with ribavirin on chronic hapatitis C. *World Journal of Gastroenterology*.

[14] Abergel A, Hezode C, Leroy V (2006). Peginterferon alpha-2b plus ribavirin for treatment of chronic hepatitis C with severe fibrosis: a multicentre randomized controlled trial comparing two doses of peginterferon alpha-2b. *Journal of Viral Hepatitis*.

[15] Ascione A, De Luca M, Tartaglione MT (2010). Peginterferon alfa-2a plus ribavirin is more effective than peginterferon alfa-2b plus ribavirin for treating chronic hepatitis C virus infection. *Gastroenterology*.

[16] Peck-Radosavljevic M, Boletis J, Besisik F (2011). Low-dose peginterferon alfa-2a is safe and produces a sustained virologic response in patients with chronic
hepatitis C and end-stage renal disease. *Clinical Gastroenterology and Hepatology*.

[17] Yan Z, Fan K, Wang Y, Fan Y, Tan Z, Deng G (2012). Changing pattern of clinical epidemiology on hepatitis C virus infection in Southwest China. *Hepatitis Monthly*.

[18] Hadziyannis SJ, Sette H, Morgan TR (2004). Peginterferon-alpha2a and ribavirin combination therapy in chronic hepatitis C: a randomized study of treatment duration and ribavirin dose. *Annals of Internal Medicine*.

[19] Weiland O, Hollander A, Mattsson L (2008). Lower-than-standard dose peg-IFN alfa-2a for chronic hepatitis C caused by genotype 2 and 3 is sufficient when given in combination with weight-based ribavirin. *Journal of Viral Hepatitis*.

[20] Zeuzem S, Feinman SV, Rasenack J (2000). Peginterferon alfa-2a in patients with chronic hepatitis C. *The New England Journal of Medicine*.

[21] Oze T, Hiramatsu N, Yakushijin T (2009). Pegylated interferon alpha-2b (Peg-IFN *α*-2b) affects early virologic response dose-dependently in patients with chronic hepatitis C genotype 1 during treatment with Peg-IFN *α*-2b plus ribavirin. *Journal of Viral Hepatitis*.

[22] Zeuzem S (2004). Heterogeneous virologic response rates to interferon-based therapy in patients with chronic hepatitis C: who responds less well?. *Annals of Internal Medicine*.

[23] Ferenci P (2012). Optimal treatment duration for patients with HCV genotype 1 infection. *Journal of Viral Hepatitis*.

[24] Ge D, Fellay J, Thompson AJ (2009). Genetic variation in IL28B predicts hepatitis C treatment-induced viral clearance. *Nature*.

[25] Popkin BM, Paeratakul S, Ge K, Fengying Z (1995). Body weight patterns among the Chinese: results from the 1989 and 1991 China health and nutrition surveys. *American Journal of Public Health*.

[26] Liao XW, Ling Y, Li XH (2011). Association of genetic variation in IL28B with hepatitis C treatment-induced viral clearance in the Chinese Han population. *Antiviral Therapy*.

[27] Yu ML, Huang CF, Huang JF (2011). Role of interleukin-28B polymorphisms in the treatment of hepatitis C virus genotype 2 infection in Asian patients. *Hepatology*.

[28] Manns MP, Wedemeyer H, Cornberg M (2006). Treating viral hepatitis C: efficacy, side effects, and complications. *Gut*.

